# Starting geometry creation and design method for freeform optics

**DOI:** 10.1038/s41467-018-04186-9

**Published:** 2018-05-01

**Authors:** Aaron Bauer, Eric M. Schiesser, Jannick P. Rolland

**Affiliations:** The Institute of Optics, University of Rochester, 275 Hutchison Road, Rochester, NY 14627 USA

## Abstract

We describe a method for designing freeform optics based on the aberration theory of freeform surfaces that guides the development of a taxonomy of starting-point geometries with an emphasis on manufacturability. An unconventional approach to the optimization of these starting designs wherein the rotationally invariant 3rd-order aberrations are left uncorrected prior to unobscuring the system is shown to be effective. The optimal starting-point geometry is created for an F/3, 200 mm aperture-class three-mirror imager and is fully optimized using a novel step-by-step method over a 4 × 4 degree field-of-view to exemplify the design method. We then optimize an alternative starting-point geometry that is common in the literature but was quantified here as a sub-optimal candidate for optimization with freeform surfaces. A comparison of the optimized geometries shows the performance of the optimal geometry is at least 16× better, which underscores the importance of the geometry when designing freeform optics.

## Abstract (web summary)

Optics that have no rotational or translation symmetry, termed freeform, have the potential to make well-corrected compact optical systems. Here, Bauer et al. approach the design of freeform optics with aberration theory and present general guidelines to design and optimize physically realizable systems.

## Introduction

Optical design is a scientific and engineering discipline where the goal is often to construct an optimal optical system that enables an optical task, such as imaging, while minimizing the errors, or optical aberrations, introduced by the optical elements by utilizing the effective degrees of freedom, such as the number and position of elements, including the aperture stop, as well as their physical shapes and materials. In another sense, optical design may have the goal of mapping an entire space of solutions that can be later ranked according to some design criteria or application. A freeform optic is defined as an optic whose surface shapes lack translational or rotational symmetry about axes normal to the mean plane. As the benefits of using freeform optics in optical design become more widely recognized and freeform optics become commonplace in optical systems, it is important to develop strategies for designing freeform systems^1–7^.

A common, first-effort method for designing with freeform optics is to vary all the coefficients that determine the freeform shape of each surface and let the raytrace optimizer determine the final coefficients and surface shapes. Surely, in the case where a favorable starting point is chosen, this method can produce a valid design, but rarely is the best design found in terms of manufacturability. Furthermore, this brute-force approach provides little physical insight and can lead to unintended consequences. For example, an overabundance of freeform terms on a surface can lead to unnecessarily large freeform departures and when low-order basis functions are used to describe an optical surface, such as the Zernike polynomials used herein, freeform departure correlates to local slope. Thus, large freeform departures and associated slopes lead to increased assembly sensitivity and difficulty in the fabrication and testing of that surface^8–11^.

Unrestricted use of freeform terms on multiple surfaces within a system can result in aberration correction degeneracy, where like-terms on separate surfaces balance one another out, resulting in a potential large increase in freeform departure for each surface with little performance gain. Methods that utilize optimization features of ray trace software have been shown to help desensitize designs^12,13^ or automate some aspects of the process^14^, but place the bulk of the responsibility on the optimization algorithm rather than aberration theory, causing a loss-of-valuable insight into the aberrations of the system. Furthermore, in the often case where a solution is not reached the only recourse is to restart with a different starting point or explore computational means to jump out of local minima.

Additionally, too seldom in unobscured reflective system design are the starting geometries analyzed from the standpoint of how much they can benefit from the use of freeform surfaces before freeform surfaces are added. The aberrations of the starting geometry determine whether the performance requirements for a given system can even be achieved within that geometry^16,17^, regardless of the amount or type of freeforms used. A common strategy for creating an obscured reflective starting design is to first design a rotationally symmetric system that is corrected for the third-order rotationally invariant aberrations (for example, a three-mirror anastigmat or a reflective triplet^18–21^) and then find a way to unobscure the system by tilting surfaces or by using a combination of a field-bias and offset aperture, without consideration of whether the induced aberrations of the newfound asymmetry can be corrected using freeform optics.

A more desirable approach is to first consider the specific unobscured geometry and its aberrations prior to the correction of third-order rotationally invariant aberrations. The ability to sufficiently correct for rotationally variant aberrations, which can be orders of magnitude greater than rotationally invariant aberrations, will determine the eventual success of the design, regardless of the state of the rotationally invariant aberrations. Then, directly target the limiting aberrations with freeform shapes in a controlled manner by only using the shapes necessary for aberration correction, which limits the overall freeform departure of a surface, thus decreasing system sensitivity, fabrication cost and testing difficulties. In addition, it reduces the required fabrication time, mitigating shape errors associated with tool wear and temperature stabilization.

In this paper, we will use the underlying aberration theory of freeform surfaces^15^ to establish a design methodology from the creation of a starting geometry with the best potential for aberration correction with freeform surfaces to the application of the final freeform terms. Using an F/3, unobscured three-mirror imager with a 200 mm aperture and 4° × 4° full field-of-view (FOV) as the design example, the developed method allows us to avoid performing a full optimization of all potential geometries by providing insight into the aberration correction potential of each geometry through an analysis of the aberrations fields of each, which can be further leveraged in subsequent optimization steps. Of the potential three-mirror geometries studied, the optimal geometry found performs at least 16× better than an alternative geometry which has been previously shown^22–24^ to yield high-performance systems, albeit with different power distributions, underscoring the importance of making an informed starting geometry decision.

## Results

### Aberrations from freeform surfaces

There are a number of ways in which one can mathematically describe the shape of a freeform surface. We chose to work with the Fringe ordering of the Zernike polynomials, with sag described mathematically by

1 $$z = \frac{{cr^2}}{{1 + \sqrt {1 - \left( {1 + k} \right)c^2r^2} }} + \mathop {\sum}\limits_j {C_jZ_j\left( {\rho ,\varphi } \right)},$$

where *c* is the curvature of the base sphere, *k* is the conic constant, *r* is the radial coordinate of the surface, *ρ* is the radial coordinate of the surface normalized by *R*_norm_, (that is, *ρ* *=* *r/R*_norm_), *φ* is the azimuthal component of the surface aperture, and *C*_*j*_ is the weight factor of the *j*^th^ Zernike term, *Z*_*j*_. This freeform representation was chosen as a result of the work done by Fuerschbach et al.^15^ on the aberration contributions from freeform surfaces described by Fringe-ordered Zernike polynomials. Using principles from nodal aberration theory^25–27^, the net aberration fields generated as a result of adding each Zernike term to an optical surface anywhere in the system are predicted and plotted in aberration full-field displays (FFDs). These predicted aberration fields are the backbone of the design method we developed because, through a simple visual comparison to the aberration FFDs produced in the optical design software, they can tell us which Zernike terms are needed on a surface at or away from the aperture stop to correct an aberration with a specific field dependence.

The standard aberration FFDs in commercial optical design software are generated by fitting the aberrated wavefront in the pupil with Zernike polynomials and plotting the resulting magnitude and orientation for each corresponding pair of Zernike terms. Because the Zernike-based aberration FFDs contain multiple orders of wavefront aberrations^28^ and the FFDs in ref. ^15^ contain only specific wavefront aberrations, visual comparisons become difficult when low-order aberrations no longer dominate. To facilitate visual comparisons between the Zernike-based aberration FFDs in design software and the predicted aberration fields, new predictive FFDs with the wavefront aberrations plotted as in a Zernike-based aberration FFD were generated (up to Z15). These new FFDs are shown in Supplementary Figs. 1-5 together with Supplementary Note 1 for the case of a freeform surface located after the stop surface, as will be the case for the imager proposed in this work. Since we now can visualize the predicted aberration field contributions from various Zernike polynomial freeform shapes in the same fashion as they are presented in the design software, we can begin the process of designing the imager.

### Freeform design methodology

In this section, we will describe the method we developed for designing freeform optical systems. Without loss-of-generality, the method will be demonstrated in the context of a three-mirror imager; however, the method and associated techniques are applicable to optical systems of all types. To help limit the design space, we chose a specific distribution of optical power over the three mirrors. In the intended application space of this imager, volume, and weight are driving constraints, so to minimize the size of the secondary and tertiary mirrors, it is preferred that the primary mirror have positive power. Placing negative power on the secondary mirror helps flatten the focal plane, and a positive tertiary mirror will create a symmetric power distribution across the primary, secondary, and tertiary mirrors that is conducive to aberration correction^18,19^. Let it be noted that the design methods that we will apply to the positive-negative-positive (PNP) case are not limited to PNP configurations, and are generally applicable to other power distributions. Additionally, without loss-of-generality of the design method, we will set the aperture stop to be located at the primary mirror surface. We will design a 200 mm aperture-class three-mirror imager operating at F/3 over a 4° × 4° full FOV, with the goal of diffraction-limited performance over the visible spectrum for a system volume of about 60 liters, measured by constructing a box that fully encloses the system. Real-ray based distortion constraints will be implemented during the optimization to allow up to 1% distortion.

### Starting geometry creation and down-selection

The creation of a geometry that maximizes the benefit from freeform surfaces and requires less overall freeform departure, which also results in lower surface slope when only low-order (here up to Z15) Zernike polynomials are considered, is an important step in reducing the sensitivity and fabrication costs of the final design. Instead of first constructing a fully obscured rotationally symmetric system that is corrected through third-order then tilting the surfaces into a planar symmetric unobscured geometry as one might do with an off-axis conic or asphere design, this method focuses on first developing the geometries that are conducive to the application of freeform surfaces.

**Fig. 1:**
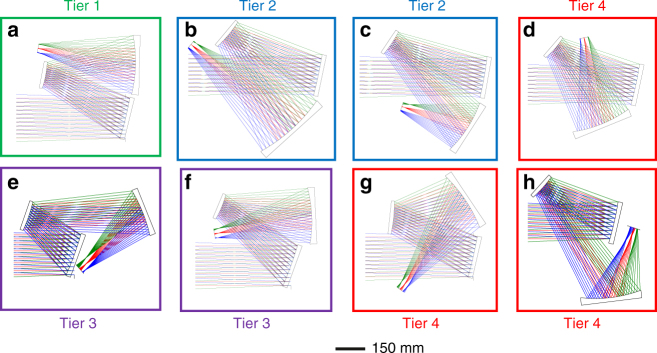
A comprehensive selection of possible geometries for unobscured positive-negative-positive (PNP) reflective imagers. Eight different geometries, differentiated by the folding directions of the mirrors, were found to be feasible for a three-mirror PNP reflective imager. Here, each system has planar symmetry about a plane parallel to the page. The different colors of the rays indicate separate points in the field-of-view (FOV). **a** The Tier 1 design has the greatest potential to be corrected using freeform surfaces; **b**, **c** the Tier 2 designs have some potential, but are limited by volume; **e**, **f** the Tier 3 designs have limited correction potential; **d**, **g**, **h** the Tier 4 designs have the least potential to be corrected. These tiers were determined by applying the three filters described in the text

For a general optical system with some freedom to choose the folding geometry, this process begins by constructing a first-order optical layout in each possible permutation of surface tilt directions and image plane locations within each permutation. The magnitudes of the surface tilts should be minimized to what is necessary to avoid obscurations. An important feature and key point is that it is not critical at this stage that the layout variables (that is, surface powers and airspaces) take specific values, but rather are chosen to hold system constraints, such as focal length and system volume. The power distribution is typically chosen based on packaging constraints, surface sizes, flat-field conditions, and FOV requirements. The following *N* + 1 step procedure may be used to help determine layout variables that provide a sufficient starting point for a general optical system with *N* surfaces. Given a target system volume, as Step 1, set all airspaces between each surface to be roughly equivalent and to take full advantage of the allowed volume. With a chosen power distribution, in Steps 2 to *N*, assign radius of curvature values to the first *N*-1 optical surfaces, where all negative surfaces have radii equal to 1.5–2.5× the chosen airspace value and all positive surfaces have radii equal to 3–4× the chosen airspace value. In the *N*^th^ + 1 step, the power of the final surface is chosen to hold the focal length of the system. It is important to reiterate that the specific values of the layout variables are not critical provided that the focal length, volume, and obscuration requirements are satisfied. Preferred values of the layout variables are determined in the optimization steps that follow developing this starting point. If other power distributions would satisfy the constraints of the design problem, this process can be repeated. The remainder of the design process will be demonstrated for the three-mirror imager, but can be adapted for a general system. A comprehensive selection of possible geometries for the imager using tilted surfaces is shown in Fig. 1, in which, the geometries have been grouped into a hierarchy of tiers based on the potential of each geometry to be corrected by freeform surfaces as predicted by methods that will be introduced below.

Freeform surfaces are not a universal solution for correcting every aberration. In fact, as noted in Supplementary Note 1, there is a specific set of aberrations that each type of Zernike polynomial freeform shape can correct. The aberration fields of each geometry will vary as a result of the tilt-direction of each mirror and the power distribution across the mirrors. As such, we can pare down the list of potential design forms by eliminating those that produce aberrations with field-dependences and magnitudes that are not readily correctable with freeform surfaces, together with additional filters related to mechanical constraints. Further, even within the group of geometries that pass such design filters, one must be careful to choose the correct metric by which to further down-select. For example, a potential misconception would be to choose the starting geometry with the least amount of total aberration (before adding freeform), as this might indicate that less freeform departure would be necessary and will produce an overall better final result. In fact, perhaps counter-intuitively, it is not generally true that the starting geometry with the least amount of aberration yields the best overall solution; instead one must delve deeper into the aberrations and find which geometry plays best with freeform surfaces.

For each potential geometry, the first step is to create a first-order unobscured layout (using only spherical surfaces) in a volume near what is desirable for the end result while minimizing the mirror tilts to the extent possible, as described earlier in this section. Then, the aberration FFDs for each potential unobscured geometry are evaluated. With spherical surfaces, the tilt-induced rotationally variant aberrations can be orders of magnitude greater than the rotationally invariant aberrations. The focus is placed on the potential for the rotationally variant aberrations to be corrected, because if they cannot be mitigated, the correction state of the rotationally invariant aberrations is inconsequential. Furthermore, at the appropriate time during optimization, the third-order rotationally invariant aberrations can be removed using a conic on each mirror^29^.

In each unobscured geometry, particular attention is paid to the rotationally variant astigmatism and coma, and to a lesser extent, defocus. These three aberration types are typically orders of magnitude larger than any others, and as such, must be corrected in an efficient manner. The specific field dependences of these large magnitude aberrations include field-constant (FC) astigmatism, field-asymmetric, field-linear (FAFL) astigmatism, FC coma, and FL medial field-curvature, which can be thought of as focal plane tilt (and will be referred to as such, hereafter). Thus, for each potential geometry, the ability to correct these types of astigmatism, coma, and defocus with freeform surfaces must be analyzed.

FC astigmatism is the easiest of the group to address since adding an astigmatism shape to any surface in the system results in the addition of FC astigmatism, or, conversely, the subtraction of FC astigmatism when correcting aberrations that are already present in the system (Supplementary Fig. 1). The correction of the next three aberrations represents a critical design step and becomes the main geometry filter. Adding a coma shape to a surface after the stop results in some amount of focal plane tilt, FAFL astigmatism, and FC coma (Supplementary Fig. 2). These are, significantly, the three major residual aberrations in most unobscured geometries after removing FC astigmatism. However, there is also a specific relative orientation between the FAFL astigmatism and FC coma that adding a coma shape to a surface after the stop can produce (Supplementary Fig. 2). This indicates that we can only fully leverage the simultaneous aberration correction power of a coma shape after the stop if the FAFL astigmatism and FC coma present in the system have the same relative orientation that is addressable by such a coma shape. For systems that present FAFL astigmatism and FC coma with relative orientation opposite to what a coma shape after the stop can address, the orientation of the FC coma must first be flipped by the over-addition of a coma shape at the stop surface, which produces only FC coma. In essence, significant aberration (FC coma) must be added before either aberration can be removed (FAFL astigmatism and FC coma, together). This results in more freeform departure of the surfaces, and increases the amount of induced higher-order aberrations generated by the existing freeform shapes. With that, we can create the first filter for the first-order unobscured geometries: the FAFL astigmatism and FC coma of the first-order unobscured geometry must have the relative orientation that is correctable by a coma surface after the stop.

Directly related to tilting optical surfaces or adding a coma shape away from the stop is the issue of focal plane tilt. Technically, the image plane can be physically tilted to eliminate any focal plane tilt aberration induced by the tilted mirrors, but that is not preferred because large image plane tilts create anamorphism of the image and result in responsivity issues for the sensor. The first filter ignored the fact that a coma shape after the stop can also create focal plane tilt. So, the second filter addresses this point: the focal plane tilt intrinsic to the tilted system must decrease as a result of using a coma shape on a surface after the stop to correct the FAFL astigmatism and FC coma.

The last filter is based on the flat-field condition and combines mechanical constraints with optical constraints. While it was stated earlier that the third-order rotationally invariant aberrations can be corrected using three conics, that does not apply to the Petzval curvature. If left uncorrected, it will limit the system before the diffraction limit can be reached. To ensure a nearly flat-field, the sum of the mirror powers must be near zero. The flat-field constraint adds a degree of complexity to the construction of an unobscured first-order layout, sometimes requiring mirrors to have fast individual F/#’s or to have extreme tilts to clear the rays. The rotationally variant aberrations (including higher-order aberrations) generated by these fast, highly tilted surfaces are large in comparison to the same geometry without the flat-field condition, and often represent unrecoverable image degradation. The third filter is thus: the specific form of the geometry that meets the flat-field condition must be well-behaved, indicated by moderate mirror tilts and speeds.

The first two filters are critical to help build an in-depth understanding of the aberrations of freeform surfaces as described in ref. ^15^. The third filter selects geometries based on more conventional optical design wisdom, but is no less useful for filtering the possible geometries. By using these three filters, we can analyze the potential of various forms within each geometry shown in Fig. 1 to yield a high-performing system. We applied all three filters to various forms within each potential geometry in Fig. 1 and found that the geometry shown in Fig. 1a was the best candidate for further optimization. This geometry was the basis for the original reflective triplet design using off-axis conics^18^ from which others have extended into the freeform regime^30–32^. Using the aberration-based analysis presented in this paper, we now have an answer to why this specific geometry is conducive to freeform surfaces. We will proceed now to describe the freeform optimization strategy using that geometry.

### Iterative freeform optimization

**Fig. 2:**
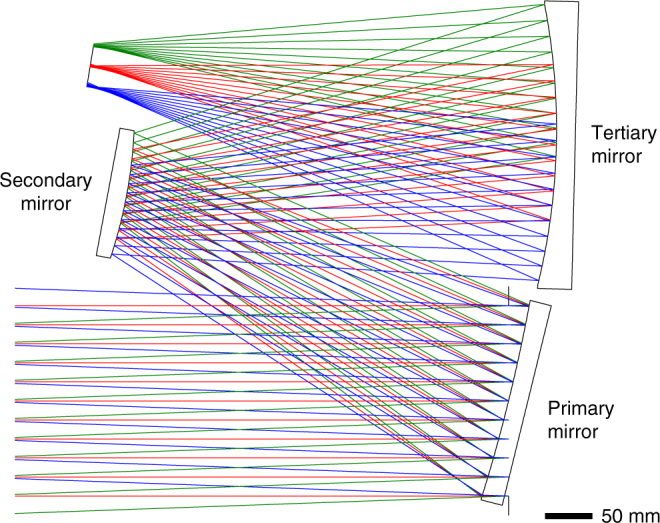
The starting design. The design with the greatest potential to be corrected using freeform surfaces was found to be Fig. 1a. The powers of the surfaces were then modified to flatten the field while holding the focal length. In its current form it consists of all spherical surfaces

With a starting geometry chosen, we can now focus on correcting the aberrations of that design. From this point, we modify the mirror radii to create a flat-field design while holding the focal length. This now represents the starting design of the system, shown in Fig. 2. We can now start to tackle the rotationally variant aberrations through the controlled addition of Zernike terms to the mirror shapes. The optimization techniques used will combine the numerical methods of the raytrace software optimizer and the aberration theory of freeform surfaces as a best practice to find the optimal set of surface shapes. Instead of slowly increasing the order of the terms added as one would do for aspheric terms or in a conventional optimization, the freeform terms are added by using the aberration theory to determine the exact freeform term and surface to modify next.

**Fig. 3:**
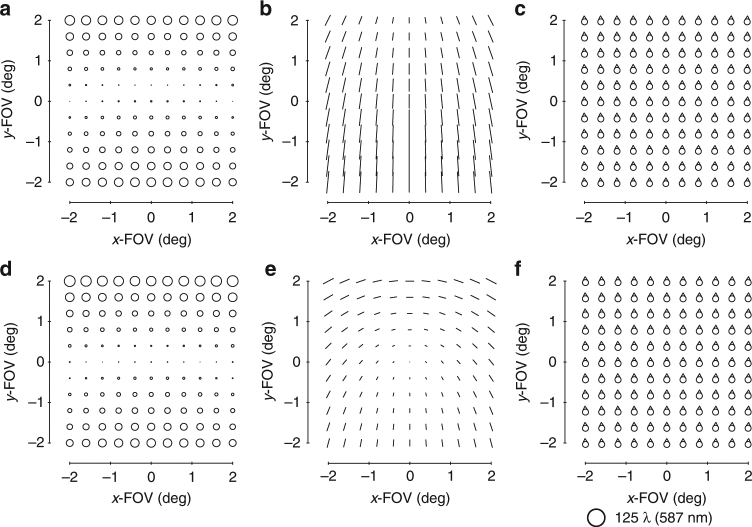
Zernike-based aberrations FFDs after an astigmatism surface. Full-field displays (FFDs) calculate and plot the magnitude and orientation of a given aberration over a set of points that sample the full FOV. The aberrations of the system shown in Fig. 2 before adding any freeform shapes are shown in the FFDs for **a** defocus (Z4), **b** astigmatism (Z5/6), and **c** coma (Z7/8). The aberrations after the addition of an astigmatism shape to the primary and tertiary mirrors are shown in the FFDs for **d** defocus (Z4), **e** astigmatism (Z5/6), and **f** coma (Z7/8). Other aberrations are negligible when plotted at this scale, which is indicated below **f**

After imposing the first-order constraints on the system, the next step is to evaluate the aberrations of the system using FFDs, as shown in Fig. 3. The two dominant aberrations types (in addition to a tilted focal plane) are astigmatism and coma. A typical, and perhaps the simplest, freeform shape we can add is an astigmatism shape, which will impart FC astigmatism into the system (Supplementary Fig. 1). This Zernike term is unique in that it only adds a single-blurring aberration regardless of whether it is at or away from the stop surface. The starting design does not have a significant amount of FC astigmatism, but there is, nonetheless, a contribution, so we use an astigmatism term to eliminate that aberration. We chose to split the freeform departure over the primary and tertiary mirrors to decrease the departure on any individual mirror and optimized the corresponding Zernike coefficients via the ray trace software. The overall aberrations are again evaluated in FFDs and are shown in Fig. 3.

**Fig. 4:**
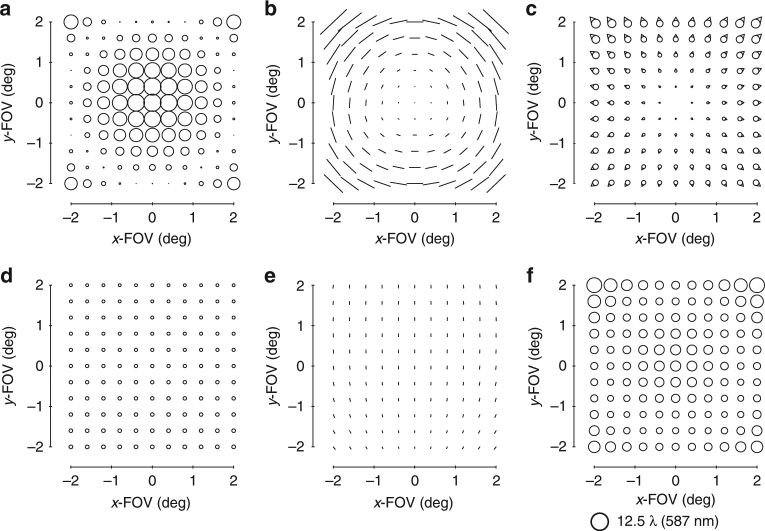
Zernike-based aberration FFDs after a coma surface. After adding a coma shape to the primary and tertiary mirrors and allowing a slight tilt of the image plane, the aberrations of the system were calculated and are shown in FFDs for **a** defocus (Z4), **b** astigmatism (Z5/6), **c** coma (Z7/8), **d** spherical aberration (Z9), **e** elliptical coma (Z10/11), and **f** root-mean-square wavefront error (RMS WFE). Note the scale decreased 10× from Fig. 3

It is clear now that there are three aberrations that limit the system: focal plane tilt, FAFL astigmatism, and FC coma. This step, in particular, is why we chose the geometry we did. By adding a coma surface to a non-stop surface (that is, the secondary or tertiary mirror), all three limiting aberrations can be reduced simultaneously (Supplementary Fig. 2). However, the ratio of the magnitudes of the three aberrations present in Fig. 3 differs from the ratios of those aberrations produced by a coma shape on either the secondary mirror or tertiary mirror. To change the system’s ratio of the FC coma to FAFL astigmatism to match what is simultaneously correctable by a coma shape on the tertiary mirror, a coma shape is added to the primary mirror (stop surface). Similar logic applies for allowing a slight physical image plane tilt (<5°). The new aberration FFDs of the system after optimizing the coma shapes via the ray trace software on the primary and tertiary mirrors are shown in Fig. 4.

**Fig. 5:**
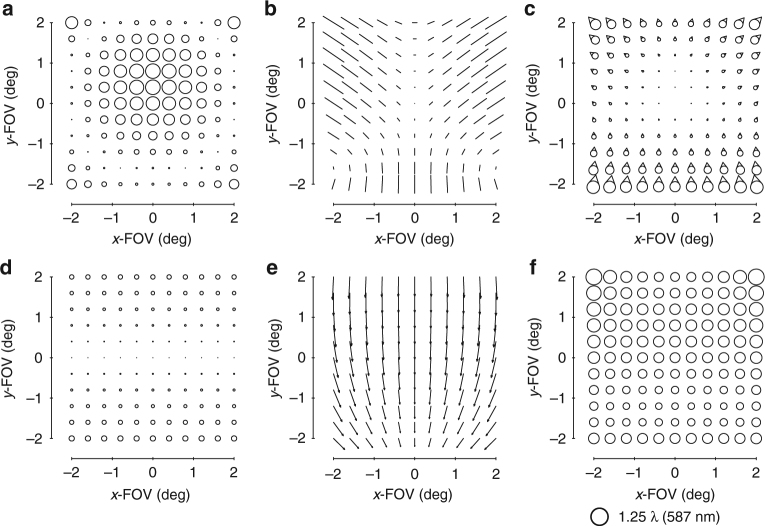
Zernike-based aberration FFDs after conics. After adding conics to all three mirrors and allowing the radii, tilts, and airspaces to vary, the aberrations of the system were calculated and are shown in FFDs for **a** defocus (Z4), **b** astigmatism (Z5/6), **c** coma (Z7/8), **d** spherical aberration (Z9), **e** elliptical coma (Z10/11), and **f** RMS WFE. Note the scale decreased 10× from Fig. 4

At this point, the system is no longer limited by rotationally variant aberrations, but rather three third-order rotationally invariant aberrations, namely astigmatism, coma, and spherical aberration. The total magnitude of these aberrations is an order of magnitude less than the rotationally variant aberrations of the starting point and are correctable by the choice of conic constants for each mirror. After adding a conic to each of the three mirrors, the rotational invariant components of the astigmatism, coma, and spherical aberration have been corrected, but it is becoming challenging to identify specific field dependencies in the FFDs due to the balancing of multiple field dependencies and the presence of both lower and higher-order aberrations. To free up the design space, we will do an optimization with the mirror radii, airspaces, and mirror tilts as free variables, while maintaining a similar overall package. The resulting system has the aberrations shown in Fig. 5. We can now continue the process of identifying and correcting the limiting aberrations using freeform surfaces.

**Fig. 6:**
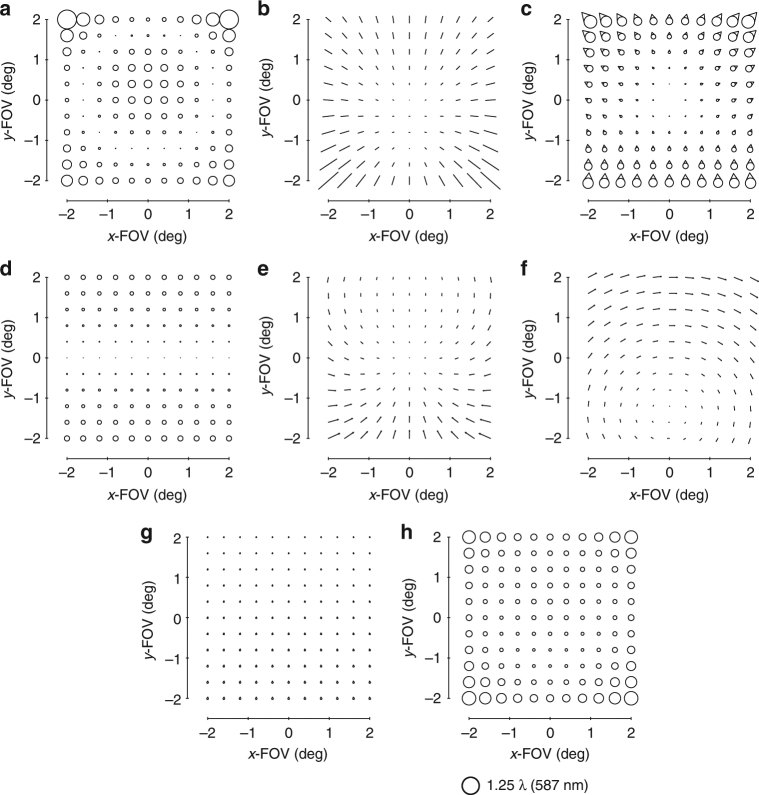
Zernike-based aberration FFDs after a trefoil surface. After adding a trefoil shape to the primary and secondary mirrors, the aberrations of the system were calculated and are shown in FFDs for **a** defocus (Z4), **b** astigmatism (Z5/6), **c** coma (Z7/8), **d** spherical aberration (Z9), **e** elliptical coma (Z10/11), **f** oblique spherical aberration (Z12/13), **g** fifth-order aperture coma (Z14/15), and **h** RMS WFE

The next limiting aberrations are identified to be field-conjugate, FL astigmatism and FC elliptical coma. By inspection, we can see that a trefoil shape away from the stop can correct these two simultaneously (Supplementary Fig. 3), so a trefoil shape is added to the secondary mirror. To allow for a full correction of these aberrations, a trefoil shape is also added to the primary mirror (stop surface). The residual aberrations are shown in Fig. 6.

**Fig. 7:**
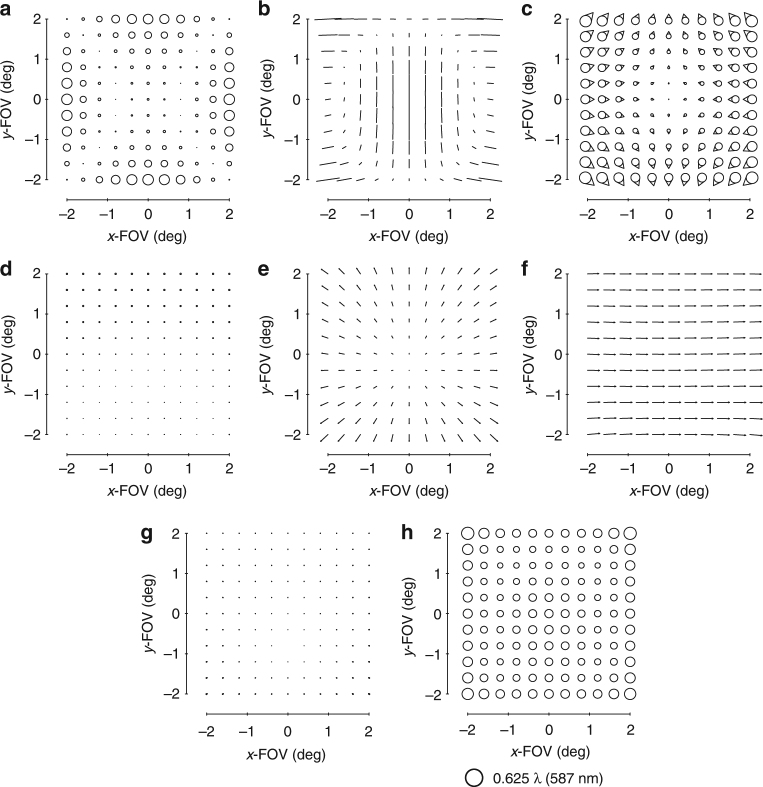
Zernike-based aberration FFDs after a fifth-order aperture coma surface. After adding a fifth-order aperture coma shape to all three mirrors and then reoptimizing the form of the geometry, the aberrations of the system were calculated and are shown in full-field displays (FFDs for **a** defocus (Z4), **b** astigmatism (Z5/6), **c** coma (Z7/8), **d** spherical aberration (Z9), **e** elliptical coma (Z10/11), **f** oblique spherical aberration (Z12/13), **g** fifth-order aperture coma (Z14/15), and **h** root-mean-square wavefront error (RMS WFE. Note the scale decreased 2× from Fig. 6

Now that we are entering the realm of the higher-order aberrations, there coexists more aberrations to correct with a single freeform shape. Inspection of Fig. 6 confirms there are at least five aberrations present (Fig. 6c–g) that can be addressed by using a fifth-order aperture coma shape on a surface located away from the stop (Supplementary Fig. 5). Like in the previous steps, the ratios of these aberrations relative to one another in the current system are not equal to the ratios that any one mirror away from the stop can correct, and because there are now five types of aberrations, we need a contribution from each of the three mirrors to maximize the correction. The system was optimized after adding a fifth-order aperture coma shape to all three mirrors. This is another point during the design process at which we chose to let the geometry vary (radii, airspaces, tilts) to find a lower minimum. The resulting aberration FFDs are shown in Fig. 7.

**Fig. 8:**
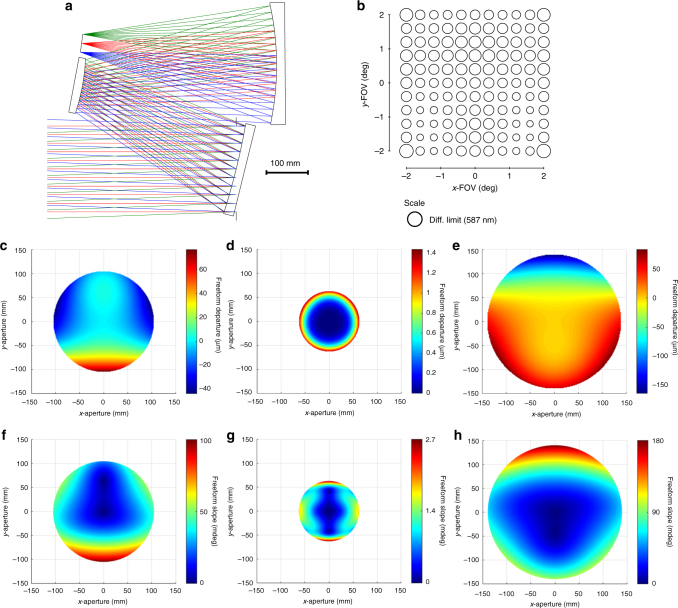
Final design properties. **a** Layout of the final design. The image plane is tilted by 1.8°. **b** Root-mean-square wavefront error (RMS WFE full-field display (FFD for the final design. All points across the FOV are diffraction limited. The peak-to-valley freeform departures from the base sphere for the **c** primary mirror, **d** secondary mirror, and **e** tertiary mirror are 122 μm, 15 μm, and 240 µm, respectively. The gradient of the freeform departure for the **f** primary, **g** secondary and **h** tertiary mirrors are 0.10°, 0.0027°, and 0.18°, respectively

The performance of the imager is now near diffraction-limited. By observing the FFDs in Fig. 7, we identify that five aberrations (Fig. 7a–c, e, f) can be corrected by using a secondary astigmatism shape on a mirror away from the stop (Supplementary Fig. 4). The secondary astigmatism shape was added to the tertiary mirror, as well as the stop surface and the system was optimized. The root-mean-square wavefront error (RMS WFE) FFD in Fig. 8b shows that the system now meets the required specification and, thus, completes the design process. For applications that would require the final geometry to be altered, such as to satisfy packaging constraints or for stray light mitigation, one can now go back and apply the alterations, followed by a similar analysis to that applied here. It is clear by this method that only the Zernike terms that were necessary for correction of the rotationally variant aberration were used and, thus, the departure for the freeform surfaces was minimized. The final surface shapes with the base sphere removed are shown in Fig. 8 together with the gradients of the freeform departures. Animations of how the surface shapes affect the aberration fields are provided in Supplementary Movie 1.

### Comparison to a conventional optimization

Conventional lens design processes have been developed extensively for rotationally symmetric systems and some of their high-level aspects are shared with the method we have described, including the need to create an enabling starting design and using aberration theory to guide the optimization. Still, the rotationally non-symmetric nature of freeform systems precludes the sole use of conventional processes, which motivated the development of the freeform design process described herein. When given a pre-determined starting geometry, however, optimization-only design strategies are blind to the configuration of the system (for example, rotationally variant or invariant) and can be implemented and evaluated for freeform systems. To compare the optimization method described in the previous section to a more conventional optimization technique where the bulk of the work is shouldered by the raytrace optimizer, we performed an additional conventional optimization on this design form.

For a fair comparison between the two methods, the design in Fig. 2 was again used as the starting design. The system was optimized with the same performance and volume constraints, but the freeform coefficients up to Z15 in Fringe were added in batches to all three mirrors without consideration of the aberrations of the system. The volume and performance requirements were satisfied in the resulting system, but the freeform departures were larger for all three mirrors—131 μm, 165 μm, and 287 µm for the primary, secondary, and tertiary mirrors, respectively. Significantly, using the aberration-based design method, the secondary mirror only required 15 µm of freeform departure; whereas, the conventional method produced a secondary mirror with 165 µm of departure—an increase of more than an order of magnitude. This demonstrates that, while a conventional optimization can yield a design that meets the performance specifications, it may not converge to the solution with the least freeform departure and associated slopes. Much remains to be explored toward the automation of lens design with freeform surfaces; whether knowledge of aberration theory or desensitization may be automatically incorporated into the optimization process or whether other methods may be sought^13,14^.

### Investigating an alternative starting geometry

To emphasize the importance of choosing the geometry that best facilitates the use of freeform optics, we will now utilize the developed design methods to optimize a similar system, but starting in a different geometry. We will demonstrate that the simple choice of the tilt directions of the mirrors dictates the degree of aberration correction possible for the system.

Consider the geometry in Fig. 1d. This geometry was found to be in the lowest tier for aberration correction potential using freeform surfaces for PNP systems. However, without using the starting-point filters to gain that knowledge, the overall geometry looks promising to use for a freeform system, and, in fact, has been commonly used in the literature^22–24^ with one important difference—a power distribution of negative-positive-positive across the three mirrors, which changes the aberration distribution, but results in larger mirrors and system volumes. Thus, upon first glance, it would be easy to assume that this geometry works well for all power distributions, but an analysis of its aberrations shows that it does not (Supplementary Figs. 6, 7)

This alternative geometry was optimized using the same methods described for the optimal geometry, however, its RMS WFE performance in a 60 L package was 65× worse than the final design in the optimal geometry in Fig. 8a. The alternative geometry was further optimized with the volume constraint loosened, yet the RMS WFE performance still lagged the optimal geometry design by 16×. Furthermore, the shapes of the surfaces required to attain this performance were significantly more aggressive at 860 μm, 2380 μm, and 2230 μm of freeform departure for the primary, secondary, and tertiary mirrors, respectively (Supplementary Fig. 8). The results of the two optimizations in this geometry give credence to our assertion that choosing the geometry that is best suited to the application of freeform optics is the first and most important aspect when choosing a starting point. Complete details on the optimization of the alternative geometry can be found in Supplementary Note 2.

## Discussion

Through the development of the design process for optical systems using freeform surfaces described by Zernike polynomials, we have seen that the most critical consideration when searching for a starting point is the geometry, which is determined by the tilt directions of the mirrors, and the method in which the potential of that geometry is evaluated. By knowing which aberrations are correctable with certain Zernike shapes either at the stop or away from the stop, an optimal starting geometry can be created. Furthermore, the correct selection of the mirror tilts influences not only the attainable aberration correction, but also the sensitivity and fabrication difficulty by minimizing the overall freeform departure of the surfaces.

The optimization steps of the design process were reported, as way of an example, for an F/3 three-mirror imager with a 200 mm aperture and 4° × 4° full FOV, resulting in a diffraction-limited solution in the visible band. The key aspect of the design process during optimization is to invoke the aberration theory of freeform surfaces to guide the design such that only the necessary freeform terms are added to the surfaces in a minimalistic manner in an effort to optimize the design for manufacture by minimizing the freeform departure and slope of each surface. This detailed starting design creation and optimization process serves to demystify the emerging field of freeform optical design.

The importance of choosing the optimal geometry was emphasized by performing an additional optimization of a geometry that has been used in the literature but in a different power distribution. The filter-based starting-point analysis predicted that with a PNP power distribution, this alternative geometry has poor potential for aberration correction with freeform surfaces. Our prediction was validated by a final design that, when compared to the final design of the optimal geometry, saw that the optimal geometry design had at least 16× better average RMS WFE.

Without loss-of-generality, we demonstrated the creation of starting points, down-selection, and optimization in the context of a three-mirror imager. Of primary importance, the methods are not limited to the case of a three-mirror imager. By limiting the system to three mirrors, the processes of starting point creation and down-selection, as well as the optimization method could be more clearly highlighted. Specifically, the aberrations for the complete system were calculated and used visually to create an enabling starting design, which is then optimized with freeform surfaces located either at or away from the pupil planes (including the aperture stop, or its conjugates in a reimaging system). The calculation of the system aberrations is independent of the number of surfaces and their locations relative to a pupil plane, thus the aberration-based design principles apply to systems with any number of surfaces, including those with intermediate images^33^. In the three-mirror imager chosen, there was one surface at a pupil plane, and two surfaces away from a pupil plane, whereas in a general case, there is no requirement for a surface to be located at a pupil plane. If none of the surfaces are at a pupil plane, the methods are still viable because, as shown in the present work and verified theoretically by Fuerschbach et al.^15^, field-constant aberrations can be managed by freeform surfaces located away from a pupil plane when the associated field-dependent aberrations can be corrected simultaneously, though this property may lead to the requirement to have more surfaces than in the case where a surface is located a pupil plane for the same design specifications.

### Data availability

The data that support the findings of this study are available from the corresponding author upon reasonable request.

## Electronic supplementary material

Supplementary Information

Description of Additional Supplementary Files

Supplementary Movie 1
