# Supplementary material for: Starting geometry creation and design method for freeform optics
Source: Nat Commun. 2018 May 1;9:1756. doi: 10.1038/s41467-018-04186-9 (PMC5931519; doi:10.1038/s41467-018-04186-9)
Supplement: Supplementary file 1 — Supplementary Information [file 41467_2018_4186_MOESM1_ESM.pdf]

Starting geometry creation and design method for freeform optics

Bauer *et al.*

Supplementary Note 1

As noted in the Results section, the aberration FFDs derived and plotted in [1] are in terms in the wavefront aberration function, *W*, meaning that each FFD plots the field dependence of a single wavefront aberration. This approach contrasts with the FFDs found within optical design software, which are generated by fitting the aberrated wavefront with Zernike polynomials and plotting the corresponding magnitudes and orientations of each Zernike term (or pair of terms) in a single FFD. As found in [2], the Zernike-based aberration FFDs for a given term contains its corresponding wavefront aberration, but also higher-order wavefront aberrations of the same azimuthal dependence. The higher-order contributions in the Zernike-based aberration FFDs make it difficult to identify the corresponding freeform term through inspection using the wavefront-based FFDs as a reference, specifically after low-order aberrations are no longer dominating. To facilitate visual comparisons, we generated Zernike-based FFDs that illustrate the aberrations produced from adding a specific Zernike shape on a surface after the stop surface. Note that as the Zernike surface is moved closer to the stop, the field-dependent aberration contributions from a Zernike shape trend towards zero. The resulting Zernike-based FFDs after adding the planar-symmetric terms of astigmatism (Z5), coma (Z8), trefoil (Z11), secondary astigmatism (Z12), and fifth-order aperture coma (Z15) as done in [1] are shown below in Supplementary Fig. 1-5. Note that only the image blurring aberrations are considered and plotted here; the effects of distortion and wavefront piston are not specifically considered here as they are dealt with separately. Distortion can either be corrected optically via ray constraints or in software [3] and the functional form of the distortions as a result of adding freeform shapes to the surfaces can be found in [1]. The wavefront aberrations (e.g. field curvature) present in each Zernike-based FFD are calculated in Gray et al. [2].

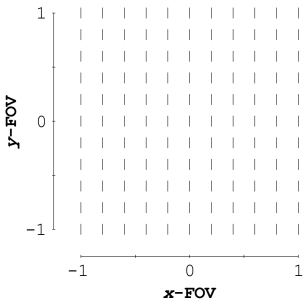

Supplementary Fig. 1. When adding an astigmatism shape (Z5) to a surface away from the stop, the only aberration generated is FC astigmatism, shown here in the astigmatism (Z5/6) Zernike-based aberration FFD.

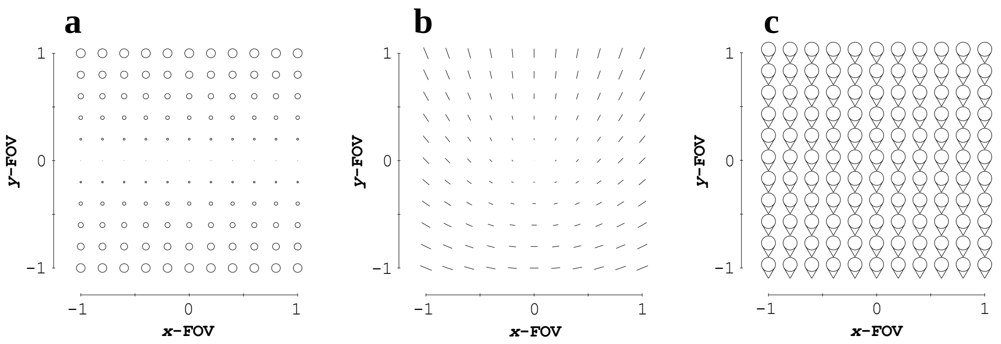

Supplementary Fig. 2. Adding a coma shape (Z8) to a surface after the stop adds three types of aberration, shown in the Zernike-based aberrations FFDs for **a** defocus (Z4), **b** astigmatism (Z5/6), and **c** coma (Z7/8). No higher-order aberrations are generated, and thus, these three FFDs match the wavefront-based FFDs in [1].

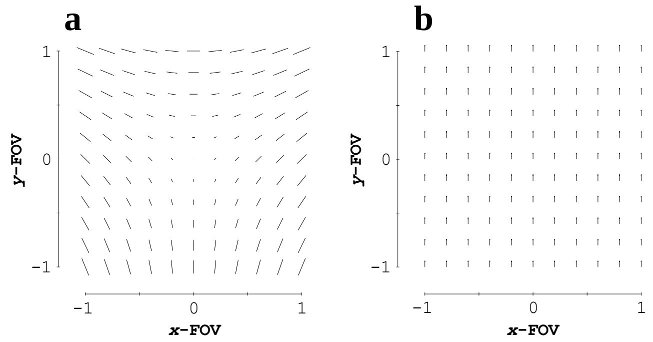

Supplementary Fig. 3. Adding a trefoil shape (Z11) to a surface after the stop adds two types of aberration, shown in the Zernike-based aberrations FFDs for **a** astigmatism (Z5/6), and **b** elliptical coma (Z10/11). No higher-order aberrations are generated, and thus, these two FFDs match the wavefront-based FFDs in [1].

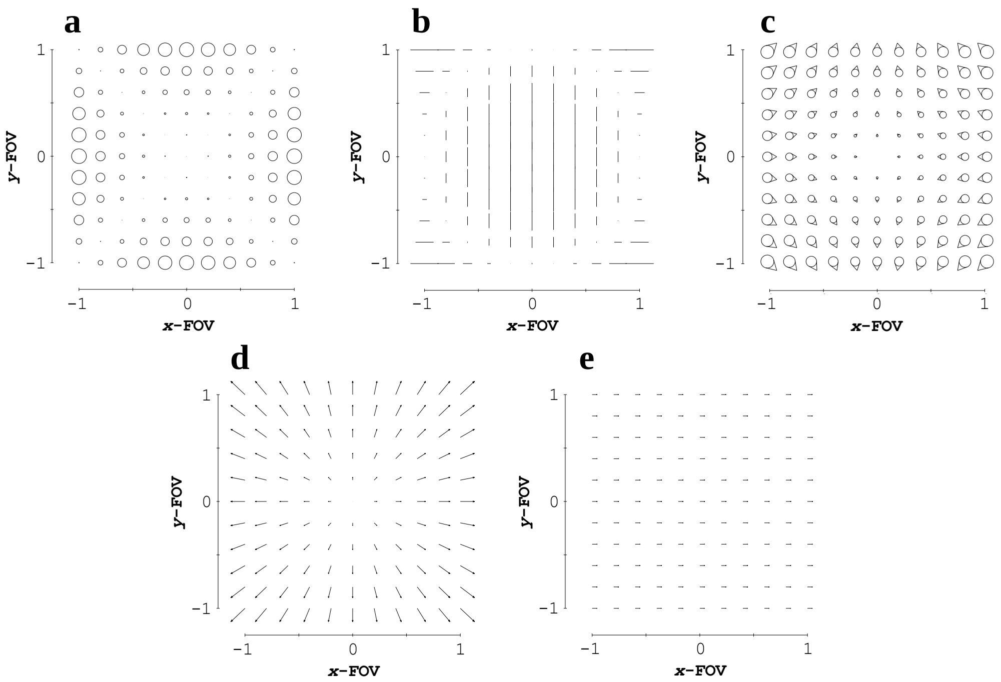

Supplementary Fig. 4. Adding a secondary astigmatism aberration shape (Z12) to a surface after the stop adds five types of aberration, shown in the Zernike-based aberration FFDs for **a** defocus (Z4), **b** astigmatism (Z5/6), **c** coma (Z7/8), **d** elliptical coma (Z10/11), and **e** oblique spherical aberration (Z12/13). FC higher-order astigmatism, W_{242}, present in **b** and the plotting routine used in CODE V for the orientation of the elliptical coma symbols in **d** are the reasons for the differences between these FFDs and the wavefront-based FFDs in [1].

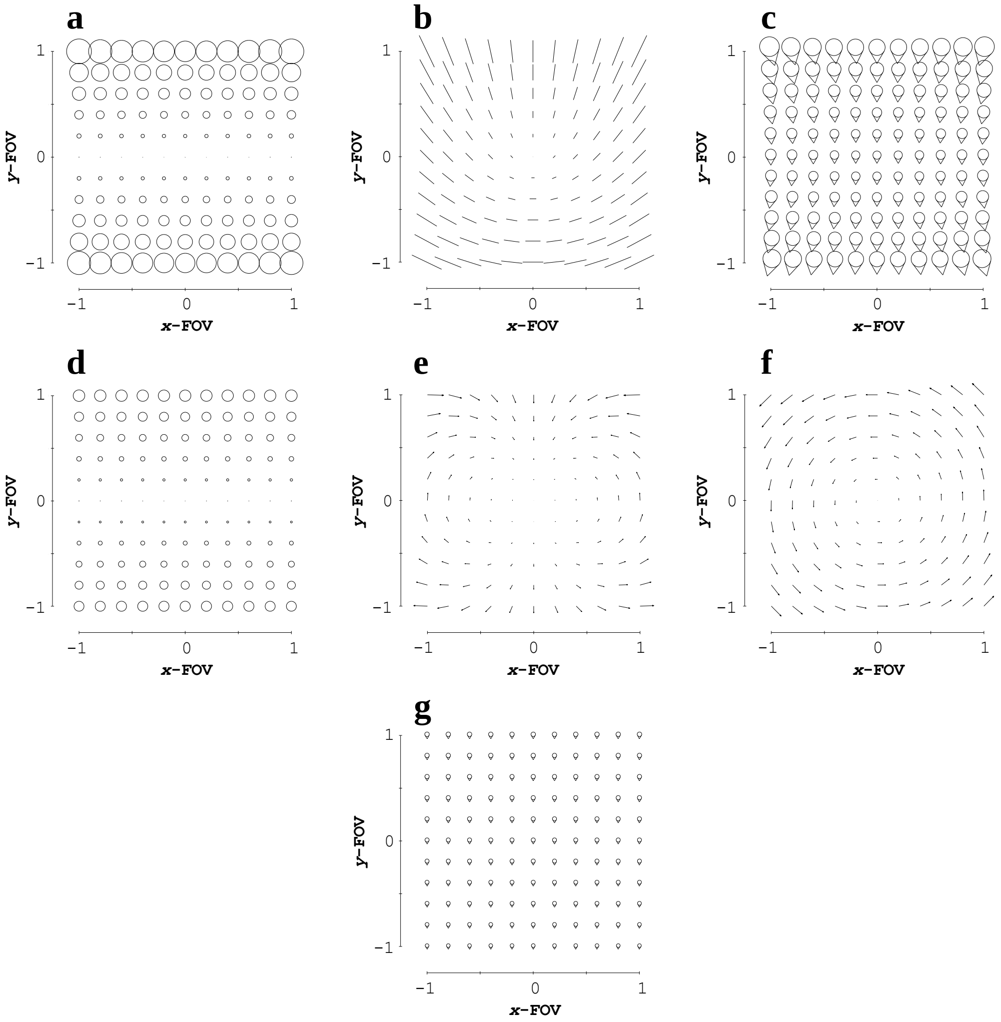

Supplementary Fig. 5. Adding a fifth-order aperture coma shape (Z15) to a surface after the stop adds seven types of aberration, shown in the Zernike-based aberrations FFDs for **a** defocus (Z4), **b** astigmatism (Z5/6), **c** coma (Z7/8), **d** spherical aberration (Z9), **e** elliptical coma (Z10/11), **f** oblique spherical aberration (Z12/13), and **g** fifth-order aperture coma (Z14/15). Higher-order contributions are present in **a** – **d** and the plotting routine used in CODE V for the orientation of the elliptical coma symbols in **e** result in the differences between these FFDs and the wavefront-based FFDs in [1]. Note that the symbols in **a** – **c** have been scaled down 2x for plotting purposes.

Supplementary Note 2

An additional optimization was performed on an alternative geometry to illustrate the importance of the mirror tilts on the performance of the system. As with the optimal geometry, the first step of the optimization is to evaluate the aberrations of the starting geometry using FFDs, shown in Supplementary Fig. 6. In comparison with the aberrations of the optimal starting geometry, we notice that the overall magnitudes of the aberrations in this geometry are greater. This means that if we could fully correct these aberrations, it would likely require more overall freeform departure for each surface. Another, and perhaps more important, difference

is the relative orientation between the FAFL astigmatism and the FC coma. Because the relative orientation between these two aberrations is not the preferred orientation, their correction potential is inferior to the Tier 1 geometry. Using a coma shape away from the stop here can correct FAFL astigmatism at the expense of adding more FC coma (or vice-versa), so the alternative strategy here is to leverage the fact that freeform shapes on the stop surface add aberrations that are constant over the FOV. So, we can use a coma shape away from the stop to correct the FAFL astigmatism, thereby increasing the FC coma. Then the residual FC coma can be corrected by a coma shape located at the stop. While those two aberrations have indeed been corrected, as shown in Supplementary Fig. 7**b,c**, the maximum amount of freeform departure introduced onto the surfaces is much greater than in the previous geometry (at a similar point in the optimization) – 1200 µm versus 250 µm, respectively. This greater departure will affect the overall sensitivity of the system, increase the fabrication difficulty, and induce higher-order aberrations to a greater extent.

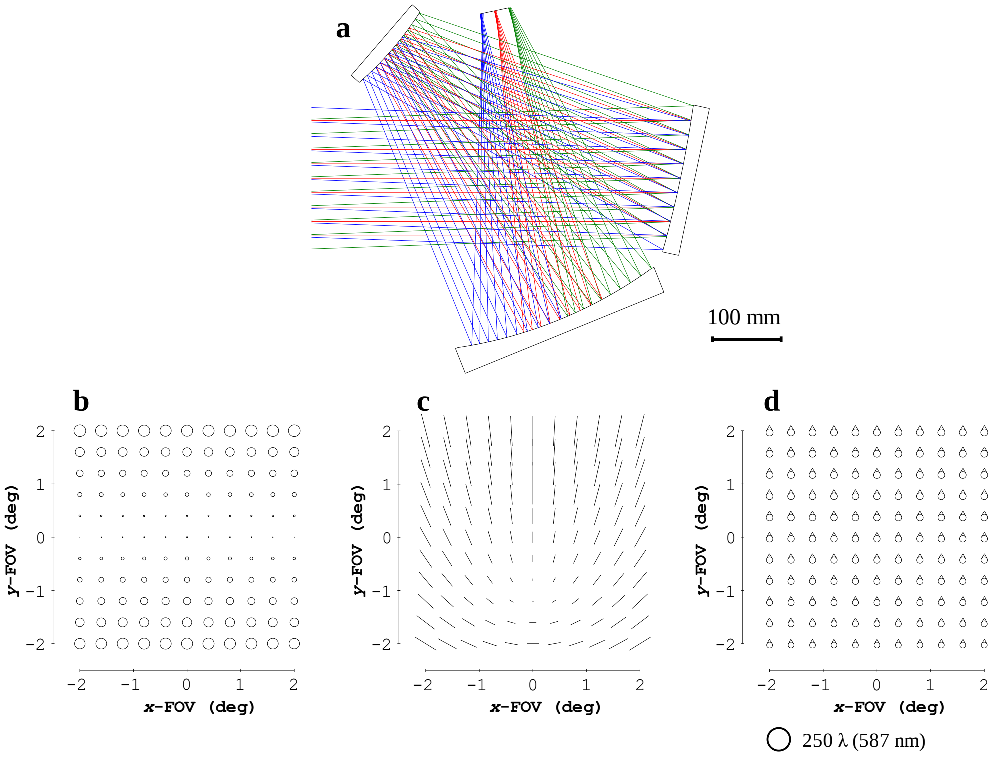

Supplementary Fig. 6. **a** The starting design consisting of all spherical surfaces in a flat-field configuration for the alternative geometry. Zernike-based aberration FFDs for the starting design showing **b** defocus (Z4), **c** astigmatism (Z5/6), and **d** coma (Z7/8) before adding a coma shape to the primary and tertiary mirrors.

Additionally, after using the two coma shapes to correct the FAFL astigmatism and the FC coma, we can see that the addition of the coma surfaces did not address the focal plane tilt of the system. Whereas the focal plane tilt was decreased simultaneously with the FAFL astigmatism and FC coma in the optimal geometry, here the coma shapes add further focal plane tilt. This requires the physical image plane to be tilted to mitigate this now limiting aberration, and quite significantly (in this case, 45°). An image plane tilt in one direction such as this creates an anamorphic image, requiring the optics to provide the opposite anamorphism in the form of large astigmatism terms on each mirror to maintain a square, low-distortion

image. Additionally, a severely tilted sensor will surely affect its responsivity. The full design process was followed, where applicable, with the specific form of the geometry constrained to be similar to the starting geometry, as this form makes efficient use of the bounding volume. However, after completing the optimization, it was seen that the performance of the system was nowhere near the specification of diffraction-limited (average RMS WFE of 32 waves), as shown in the RMS WFE FFD of Supplementary Fig. 8**a**.

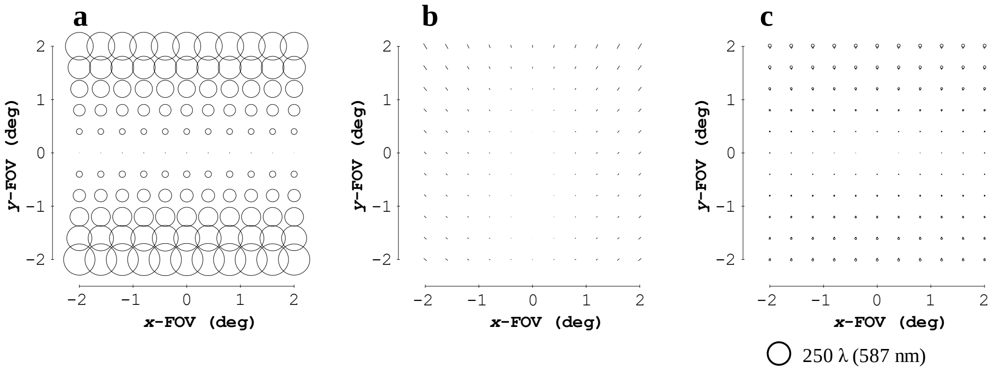

Supplementary Fig. 7. After adding a coma surface to the primary tertiary mirrors, the FAFL astigmatism and FC coma can be removed, albeit, inefficiently. However, the focal plane tilt increased by a factor of 2.5x. The aberrations have been plotted in Zernike-based aberration FFDs for **a** defocus, **b** astigmatism, and **c** coma.

Another optimization was completed, but this time with the constraints on the specific form of the geometry removed (but still using the same mirror-tilt directions). The final system, shown together with its RMS WFE FFD in Supplementary Fig. 8**c,d**, performs 16x worse than the design with the optimal geometry (average RMS WFE). The freeform departures from the best-fit sphere for the primary, secondary, and tertiary mirrors are 860 µm, 2380 µm, and 2230 µm, respectively, and, yet, the system still does not perform to specification. The geometry into which this system was folded simply does not produce the types of aberrations that are readily correctable with freeform surfaces. As can be seen in the final design, the curvatures of the primary and secondary mirrors were essentially eliminated to remove the impact of their tilts from the overall aberrations. Having power on only a single mirror causes rotationally invariant versions of astigmatism, coma, and field-curvature to become limiting. The results of the two optimizations in this geometry give credence to our assertion that choosing the geometry that is best suited to the application of freeform optics is the first and most important choice when picking a starting point.

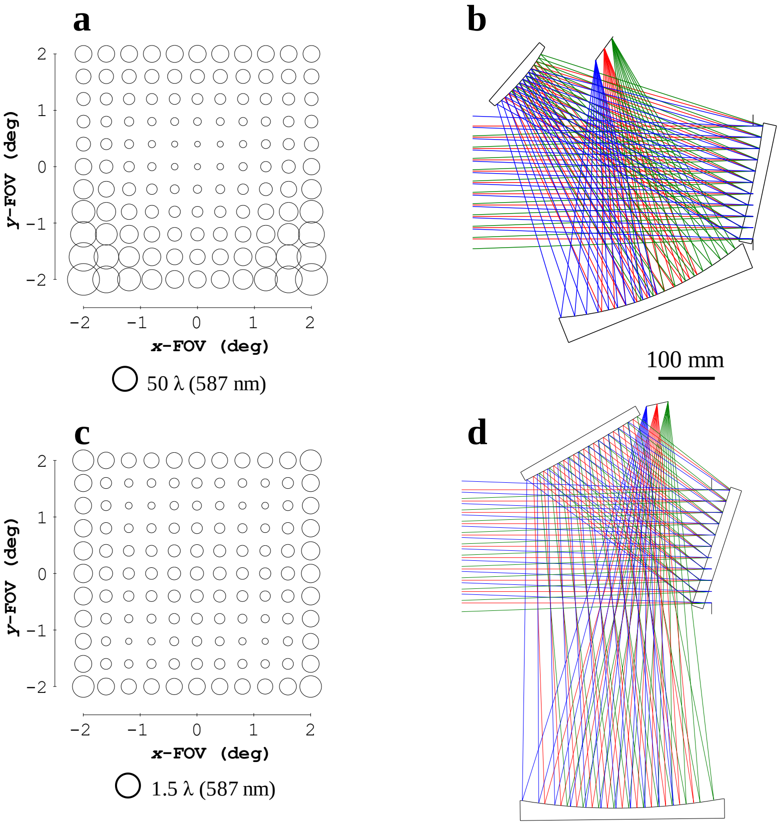

Supplementary Fig. 8. **a** RMS WFE FFD for the **b** final design in the alternative geometry with geometry constraints enforced. The performance attained by this system is 65x less than for the optimal geometry. **c** RMS WFE FFD for the **d** final design in the alternative geometry with the geometry constraints relaxed. The performance attained by this system is 16x less than for the optimal geometry.

Supplementary References

1) K. Fuerschbach, J. P. Rolland, and K. P. Thompson, "Theory of aberration fields for general optical systems with freeform surfaces," Optics Express **22**, 26585-26606 (2014).

2) R. W. Gray, C. Dunn, K. P. Thompson, and J. P. Rolland, "An analytic expression for the field dependence of Zernike polynomials in rotationally symmetric optical systems," Optics Express **20**, 16436-16449 (2012).

3) A. Bauer, S. Vo, K. Parkins, F. Rodriguez, O. Cakmakci, and J. P. Rolland, "Computational optical distortion correction using a radial basis function-based mapping method," Optics Express **20**, 14906-14920 (2012).
