# Supplementary material for: Starting geometry creation and design method for freeform optics
Source: Nat Commun. 2018 May 1;9:1756. doi: 10.1038/s41467-018-04186-9 (PMC5931519; doi:10.1038/s41467-018-04186-9)
Supplement: Supplementary file 2 — Description of Additional Supplementary Files [file 41467_2018_4186_MOESM2_ESM.pdf]

Description of Additional Supplementary Files

Supplementary Movie 1: Each step of the design process is animated, showing the limiting aberration fields and how they change as a result of adding specific freeform surfaces to the system, which is also animated.
